# Telepsychiatry and face-to-face psychiatric consultations during the first year of the COVID-19 pandemic in Australia: patients being heard and seen

**DOI:** 10.1177/10398562211046301

**Published:** 2021-11-23

**Authors:** Jeffrey CL Looi, Stephen Allison, Tarun Bastiampillai, William Pring, Stephen R Kisely

**Affiliations:** Academic Unit of Psychiatry and Addiction Medicine, The Australian National University Medical School, Canberra Hospital, Canberra, ACT, Australia; Private Psychiatry, Canberra, ACT, Australia; Consortium of Australian-Academic Psychiatrists for Independent Policy and Research Analysis (CAPIPRA), Canberra, ACT, Australia; Consortium of Australian-Academic Psychiatrists for Independent Policy and Research Analysis (CAPIPRA), Canberra, ACT, Australia; College of Medicine and Public Health, Flinders University, Adelaide, SA, Australia; Consortium of Australian-Academic Psychiatrists for Independent Policy and Research Analysis (CAPIPRA), Canberra, ACT, Australia; College of Medicine and Public Health, Flinders University, Adelaide, SA, Australia; Department of Psychiatry, Monash University, Clayton, VIC, Australia; Monash University, and Centre for Mental Health Education and Research at Delmont Private Hospital, Melbourne, VIC, Australia; Private Psychiatry, Melbourne, VIC, Australia; School of Medicine, The University of Queensland, Princess Alexandra Hospital, Woolloongabba, Brisbane, QLD, Australia; Departments of Psychiatry, Community Health and Epidemiology, Dalhousie University, Halifax, Nova Scotia, Canada

**Keywords:** COVID-19, telepsychiatry, telehealth, psychiatrist, private practice

## Abstract

**Objective:**

The Australian federal government introduced additional Medicare Benefits Schedule (MBS) telehealth-items to facilitate care by private psychiatrists during the COVID-19 pandemic.

**Method:**

We analysed private psychiatrists’ uptake of video and telephone-telehealth, as well as total (telehealth and face-to-face) consultations for April 2020–April 2021. We compare these to face-to-face consultations for April 2018–April 2019. MBS-Item service data were extracted for COVID-19-psychiatrist-video- and telephone-telehealth item numbers and compared with face-to-face consultations for the whole of Australia.

**Results:**

Psychiatric consultation numbers (telehealth and face-to-face) were 13% higher during the first year of the pandemic compared with 2018–2019, with telehealth accounting for 40% of this total. Face-to-face consultations were 65% of the comparative number of 2018–2019 consultations. There was substantial usage of telehealth consultations during 2020–2021. The majority of telehealth involved short telephone consultations of ⩽15–30 min, while video was used more, in longer consultations.

**Conclusions:**

Private psychiatrists and patients continued using the new telehealth-items during 2020–2021. This compensated for decreases in face-to-face consultations and resulted in an overall increase in the total patient contacts compared to 2018–2019.

In response to concerns about possible mental health consequences of the pandemic and to improve patient access during periodic lockdowns, the federal government introduced additional Medicare Benefits Schedule (MBS) telehealth-items for private psychiatrists in March 2020.^1^ This resulted in an expansion of telehealth in the subsequent 12 months. Private psychiatric practice is mainly office-based, providing 50%−60% of specialist psychiatric care.^2^ Consequently, telehealth was rapidly adopted, especially by April 2021 when more MBS telehealth-items were available and free of billing restrictions.^3^

However, the COVID-19 pandemic continues to evolve, with a wave of the Delta variant in both Australia and overseas from mid-2021, and thus the possibility of a long-term need for telepsychiatry.^4^ In addition, telepsychiatry allows greater flexibility and may be patients’ preferred option under certain circumstances. Therefore, we analysed the ongoing use of telehealth and face-to-face consultations by psychiatrists during the first year of COVID-19 public health measures in Australia in comparison to the 12-month period between April 2018 and April 2019.

## Methods

MBS-Item Service data were extracted from the Services Australia Medicare Item Reports (http://medicarestatistics.humanservices.gov.au/statistics/mbs_item.jsp) for psychiatrist practice office-based face-to-face consultations, COVID-19 video- and telephone-telehealth consultations for April 2020–April 2021 (2020–21 hereinafter) in Microsoft Excel format, and transferred to a purpose-built Excel database and analysed (totals, proportions, percentages). We extracted, as a baseline comparator, face-to-face consultation data from April 2018 to April 2019 (2018–19 hereinafter; Table 1). We excluded existing rural video-telepsychiatry items as they were not comparable to the current COVID-19 items.

**Table 1.** Overall data summary

In-Person	F2F 2020–21	Video Item	VideoTele20-21	Telephone Item	TeleTele20-21	F2F 18–19	F2F20-21/18-19%	Vid+Tel2020-21	Vid+Tel+ F2F2020-21	Vid/TotalTeleh2020-21	TotalTelheal+F2F20-21/F2F2018-19%	Telehealth20-21/TotalTelehealthF2F20-21%	TotalTelehealth20-21/F2F2018-19%
289	180	92434	67	92474	9	349	51.58	76	256	88.16	73.35	29.69	21.78
291	33,179	92435	5,943	92475	5,957	45,837	72.38	11,900	45,079	49.94	98.35	26.40	25.96
293	5,444	92436	873	92476	2,665	9,597	56.73	3,538	8,982	24.67	93.59	39.39	36.87
296	114,050	92437	17,450	92477	5,330	127,482	89.46	22,780	136,830	76.60	107.33	16.65	17.87
300	17,846	91827	5,885	91837	35,256	27,897	63.97	41,141	58,987	14.30	211.45	69.75	147.47
302	153,538	91828	34,598	91838	120,972	232,671	65.99	155,570	309,108	22.24	132.85	50.33	66.86
304	460,295	91829	101,180	91839	201,557	649,219	70.90	302,737	763,032	33.42	117.53	39.68	46.63
306	385,312	91830	168,489	91840	116,587	651,380	59.15	285,076	670,388	59.10	102.92	42.52	43.76
308	22,399	91831	5,387	91841	5,175	37,733	59.36	10,562	32,961	51.00	87.35	32.04	27.99
342	24,326	92455	2,896	92495	374	37,186	65.42	3,270	27,596	88.56	74.21	11.85	8.79
344	176	92456	79	92496	30	617	28.53	109	285	72.48	46.19	38.25	17.67
346	1,977	92457	1,107	92497	165	4,705	42.02	1,272	3,249	87.03	69.05	39.15	27.04
348	26,667	92458	2,412	92498	3,716	29,079	91.71	6,128	32,795	39.36	112.78	18.69	21.07
350	18,650	92459	1,747	92499	1,538	21,727	85.84	3,285	21,935	53.18	100.96	14.98	15.12
352	41,115	92460	3,937	92500	9,030	42,592	96.53	12,967	54,082	30.36	126.98	23.98	30.44
**TOTAL**	**1,305,154**		**352,050**		**508,361**	**1,918,071**	**68.05**	**860,411**	**2,165,565**	**40.92**	**112.90**	**39.73**	**44.86**

*Note.*

Face-to-Face: Psychiatrist Office-Based Face-to-Face MBS-Item-Number:

• New patient assessment items are telehealth items for new patients for an individual psychiatrist corresponding to face-to-face consultations: 289 (assessment of new patient with autism), 291 (comprehensive assessment and 12-month treatment plan), 293 (review of 291 plan) and 296 (new patient for a psychiatrist or patient not seen in last 2 calendar years).

• Standard office-based consultation items are time-based items for current and ongoing patients for an individual psychiatrist, corresponding to face-to-face consultations: 300 (<15 min), 302 (15–30 min), 304 (30–45 min), 306 (45–75 min) and 308 (75 min plus).

• Group psychotherapy provided by a psychiatrist item equivalents: 342 (group psychotherapy 1 h plus of two–nine unrelated patients), 344 (group psychotherapy 1 h plus of family of three patients) and 346 (group psychotherapy 1 h plus of family group of two patients).

• Items for interview of a person other than the patient, by a psychiatrist, for the care of the patient: 348 (initial diagnostic evaluation, 20–45 min), 350 (initial diagnostic evaluation, 45 min plus) and 352 (20 min plus, not exceeding four consultations).

Totals and percentages were calculated for combined video and telephone telehealth as a proportion April 2020–-April 2021face-to-face consultations, as well as the combined-total of video-telephone telehealth and face-to-face consultations for April 2020–April 2021. Video telehealth consultations were calculated as a percentage of total of video-telephone telehealth consultations for April 2020–April 2021. The sum-total of video-telephone telehealth and face-to-face consultations for April 2020–April 2021 was calculated as a percentage of April 2018–April 2019 face-to-face consultations:

• F2F 20-21: Face-to-Face consultations for April 2020-April 2021: (count).

• Video Item: Psychiatrist Video-telehealth MBS-Item-Number.

• VideoTele20-21: Psychiatrist Video-telehealth MBS-Item-Number Services (count).

• Telephone Item: Psychiatrist Telephone-telehealth MBS-Item-Number.

• TeleTele20-21: Psychiatrist Telephone-telehealth MBS-Item-Number Services (count).

• F2F 18-19: Face-to-Face consultations for April 2018- April 2019: (count).

• F2F20-21/18-19%: [(F2F 20-21)] divided by (F2F 18-19)] multiplied by 100: (percentage).

• Vid+Tel20-21: [(VideoTele20-21)] plus (TeleTele20-21)]: (count).

• Vid+Tel+F2F20-21: [(VideoTele20-21) plus (TeleTele20-21) plus (F2F 20-21)]: (count).

• Vid/TotalTeleh20-21%: {VideoTele20-21 divided by Vid+Tel20-21} multiplied by 100: (percentage).

• TotalTelheal+F2F2020/F2F2019%: [(Vid+Tel+F2F2020) divided by (F2F 2019)] multiplied by 100: (percentage).

• Telehealth20-21/TotalTelehealthF2F20-21%: [(Vid+Tel20-21) divided by (Vid+Tel+F2F20-21)] multiplied by 100: (percentage).

• Telehealth20-21/TotalTelehealth+F2F20-21%: [(Vid+Tel20-21) divided by (Vid+Tel+F2F20-21)] multiplied by 100: (percentage).

• TotalTelehealth20-21/F2F18-19%: [(Vid+Tel20-21) divided by (F2F 18-19)] multiplied by 100: (percentage).

## Results

### Overall findings

**Figure 1.**
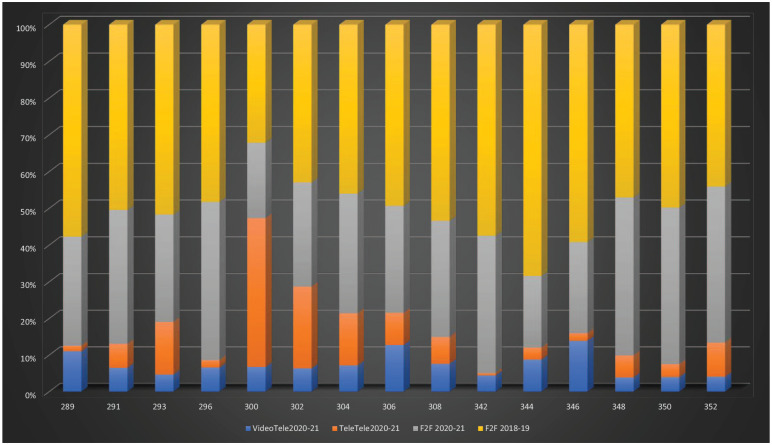
**April 2020–April 2021 and April 2018–April 2019 individual psychiatrist MBS-Item usage by modality and year**. *Note*. MBS-equivalent item numbers on y-axis; percentage of total consultations on x-axis; VideoTele20-21: Video-telehealth count April 2020–April 2021; TeleTele20-21: Tele-telehealth count April 2020–April 2021; F2F20-21: Face-to-Face consultations April 2020–April 2021: (count); F2F 18-19: Face-to-Face consultations April 2018–April 2019 (count).

For 2020–21, the total combined use of telehealth and face-to-face consultations increased by 13% compared to the equivalent period in 2018–19 (see Table 1 and Figure 1).

This increase included a substantial reduction in face-to-face consultations, which were only 65% of those 2018–19. When used, face-to-face consultations were most frequently used for new patient assessments (Items: 289, 291, 293, 296), or for longer consultations for existing patients of up to 30 min (Items: 304, 306, 308).

**Figure 2.**
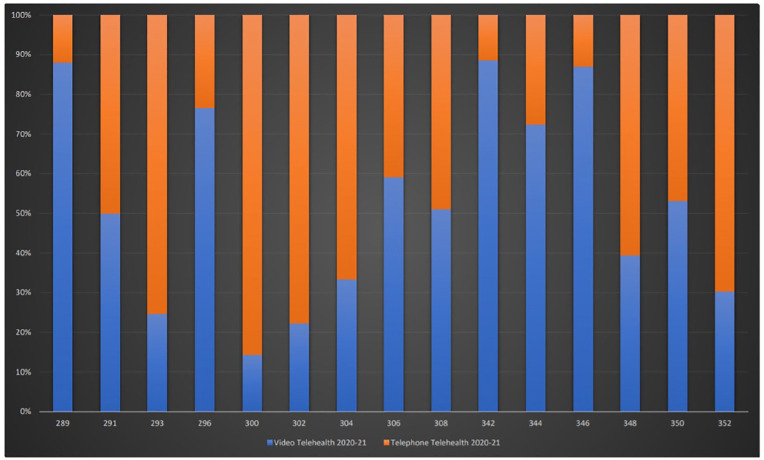
**April 2020–April 2021 video vs telephone telehealth**. *Note*. MBS-equivalent item numbers on y-axis; percentage of total consultations on x-axis; Video Telehealth 20-21: Video telehealth consultations April 2020–April 2021; Tele Telehealth 20-21: Telephone telehealth consultations April 2020–April 2021.

Video and telephone telehealth constituted 40% of the combined total of telehealth and face-to-face consultation for 2020–21 (Figure-1). Telephone-telehealth was predominantly used for shorter consultations (⩽15–30 min) with correspondingly greater video-telehealth usage in longer consultations (⩾30–75 min; Figure 2). Telephone consultations remained prominent, especially for shorter consultations, obviating travel time for appointments.

### COVID-19 psychiatrist MBS telehealth item usage

#### New patient assessment by telehealth

The telehealth alternatives of MBS items for new patients were used less frequently than the face-to face equivalents in the previous year at 18%−37% of the combined total of (telehealth and face-to-face) consultations for 2018–19 depending on the item (Table 1, Figures 1 and 2).

- *In terms of specific items, we found the following*: Telehealth new patient assessments for autism spectrum disorders (289-equivalents) were 22% of the pre-COVID-19 face-to-face consultations 2018–19, with video-telehealth used in 88% of these consultations.
- Telehealth new patient assessment and 12-month treatment plans (291-equivalents) were 26% of 2018–19 face-to-face consultations, with video-telehealth used in 50% of telehealth consultations.
- Telehealth follow-up assessment of previously new patient seen for a 12-month treatment plan (293-equivalents – patients previously seen using a 291-equivalent) were 37% of 2018–19 face-to-face consultations, with video-telehealth used in 25% of these consultations.
- Telehealth new patient assessment items without the requirement for a 12-month treatment plan (296-equivalents) were 18% of 2018–19 face-to-face consultations, with video-telehealth used in 77% of these consultations.

The combined total of (telehealth and face-to-face) new patient assessments for 2020–21 roughly equalled 2018–19 face-to-face consultations, from the lowest of 73% for assessments for autism (289) to 94%−107% for new assessments and reviews (291,293,296).

#### Standard office-based consultations for ongoing care

Much of the overall increase in telehealth consultations comprised of item 300-equivalents, that is, consultations <15 min, representing an 147% increase above the 2018–19 face-to-face consultations. For 300-equivalent-telehealth-consultations, >86% were via telephone (Table 1, Figures 1 and 2).

- *In terms of specific items, we found the following*: Telehealth contacts for 15–30 min (302-equivalents) were ⩾67% of the face-to-face consultations for 2018–19. Of these consultations, 78% were by telephone.
- Telehealth for 30–45 min (304-equivalents) was 47% of the face-to-face consultations for 2018–19, with video used in 33% of consultations.
- Telehealth for 45–75 min (306-equivalents) was 44% of the face-to-face consultations for 2018–19, and use of video was 59% of all telehealth.
- Telehealth for 75 min plus (308-equivalents) was 28% of the face-to-face consultations for 2018–19, with video used in 51% of telehealth consultations.
- Telehealth-consultations with – a person other than the patient to provide ongoing care – (348,350,352-equivalents) were used for only between 15% and 30% of the face-to-face equivalents 2018–19.

Fifteen-to-thirty-minute telehealth consultations (300-302-equivalents) represented most of the telehealth usage. Less telehealth was used for longer consultations.

The combined total of (telehealth and face-to-face) standard office-based consultations for 2020–21 equalled/exceeded 2018–19 consultations, from the lowest of 87% for >75 min (308) to range from 103% to 211% for items 300-306.

#### Group psychotherapy psychiatrist telehealth items

Group psychotherapy telehealth consultations were rarely used, possibly because face-to-face consultations were preferred for this type of intervention (Table 1, Figures 1 and 2). The combined total of (telehealth and face-to-face) group psychotherapy for 2020–21 was 46%−74% of the 2018–19 face-to-face psychotherapy consultations indicating a decline in overall MBS-rebated group psychotherapy.

## Discussion

Telehealth services have formed an important part of psychiatric care in the first year of the COVID-19 pandemic. This resulted in a 13% increase in the overall combined level of service (telehealth and face-to-face combined) compared to face-to-face-only office-based consultations in 2018–19. The new telepsychiatry services more than made up for the shortfall of face-to-face consultation that was only 65% of the 2018–19 level. As patients largely pay for their private psychiatrist COVID-19-MBS-consultations, use is likely to be driven, at least partially, by either convenience or demand. The use of shorter telephone telehealth represented an efficient way for patients to consult psychiatrists during the pandemic.

The overall usage of telepsychiatry is consistent with the international pattern in developed countries, where there was a rapid and significant uptake following similar administrative changes in Australia.^5^ Ongoing telepsychiatry usage through 2020–2021 is possibly both due to health provider^6^ and patient satisfaction.^7^ For instance, patient surveys indicate that up to 64% either agreed or strongly agreed they would consider telepsychiatry post COVID-19,^7^ while 64% of mental healthcare providers wished to use telepsychiatry for at least 25% of their patient case load.^6^ There are also indications that telepsychiatry consultations are more likely to be attended (odds ratio of 6.68) compared to an in-person visit during COVID-19, and a telepsychiatry consultation had 3.00 times the odds of being attended than a pre-COVID-19 in-person visit, in data from New York City Health and Hospitals (11 hospital-based and four free-standing clinics).^8^

Telephone-telehealth was generally used for shorter consultations (⩽15–30 min). Other work suggests that patients 45 years and older may prefer telephone to video consultations.^9^ From a patient perspective, telepsychiatry has been rated as good or excellent by 82.2% using video, and 81.5% using telephone.^7^ Up to 66% of mental healthcare providers rated telephone telepsychiatry as excellent or good, while 73% of providers rated similarly for video-telepsychiatry.^6^ Provision of in-depth care during new patient assessment, as well as for ongoing patients, and longer consultations (⩾30–75 min) involved substantial video-telehealth, perhaps reflecting increasing experience and confidence with telehealth technology.

From the patient perspective, the advantages of telepsychiatry have been identified as a lack of need to commute (46.1%) as well as flexible scheduling/rescheduling (45.5%), while disadvantages were missing the clinic/hospital (30.7%) and less connection to their doctor/therapist (20.6%), with only 8.9% expressing concern about confidentiality/privacy.^7^ The advantages of telepsychiatry identified from a mental healthcare provider perspective included timely starts (69%) and flexible scheduling/rescheduling (77%), while challenges were problems with conferencing devices (52%), lack of rapport (46%), technical problems (39%) and a low level of confidentiality/privacy concerns (16%).^6^

Notably, there was overall decline in group psychotherapy provided by all modalities in 2020–21 relative to 2018–19. Similarly, there was a relative decline in *interview of a person other than a patient.* This indicates that the pandemic was not conducive to such therapy or the involvement of relatives and carers.

## Limitations

Our results on the uptake of telehealth may have slightly underestimated the true demand given there was a phased introduction of COVID-19-telehealth-items with restrictions to bulk-billing until 20 April 2020. In addition, given that the increases in overall consultations were driven by telehealth, we do not know whether this was due to greater need, increased demand or easier access. Finally, the use of telepsychiatry may be limited by technical issues or not be appropriate for all patients.^10^

## Conclusions

Future studies are needed into the relative proportions of newly referred and existing patients on the face-to face and telehealth groups, as well as their demographic details. These data should be supplemented by local information on service outcomes, satisfaction with services, and patient/psychiatrist consultation preferences. Further study is required on the usefulness of short telephone conversations.

Ongoing use of COVID-19-telehealth-items, by patients and psychiatrists indicates their complementary utility to face-to-face care, now and post-COVID-19. Telepsychiatry has been seen and heard as a healthcare modality, especially in preparation for similar future pandemics.^4^
